# Multi-Modality Medical Image Fusion Using Convolutional Neural Network and Contrast Pyramid

**DOI:** 10.3390/s20082169

**Published:** 2020-04-11

**Authors:** Kunpeng Wang, Mingyao Zheng, Hongyan Wei, Guanqiu Qi, Yuanyuan Li

**Affiliations:** 1 School of Information Engineering, Southwest University of Science and Technology, Mianyang 621010, China; wkphnzk@163.com; 2 Robot Technology Used for Special Environment Key Laboratory of Sichuan Province, Mianyang 621010, China; 3 College of Automation, Chongqing University of Posts and Telecommunications, Chongqing 400065, China; ZMYzhengmingyao@126.com (M.Z.); weihy12@126.com (H.W.); 4 Computer Information Systems Department, State University of New York at Buffalo State, Buffalo, NY 14222, USA; qig@buffalostate.edu

**Keywords:** medical image fusion, convolutional neural network, image pyramid, multi-scale decomposition

## Abstract

Medical image fusion techniques can fuse medical images from different morphologies to make the medical diagnosis more reliable and accurate, which play an increasingly important role in many clinical applications. To obtain a fused image with high visual quality and clear structure details, this paper proposes a convolutional neural network (CNN) based medical image fusion algorithm. The proposed algorithm uses the trained Siamese convolutional network to fuse the pixel activity information of source images to realize the generation of weight map. Meanwhile, a contrast pyramid is implemented to decompose the source image. According to different spatial frequency bands and a weighted fusion operator, source images are integrated. The results of comparative experiments show that the proposed fusion algorithm can effectively preserve the detailed structure information of source images and achieve good human visual effects.

## 1. Introduction

In the clinical diagnosis of modern medicine, various types of medical images play an indispensable role and provide great help for the diagnosis of diseases. To obtain sufficient information for accurate diagnosis, doctors generally need to combine multiple different types of medical images from the same position to diagnose the patient’s condition, which often causes great inconvenience. If multiple types of medical images are only analyzed by doctor’s space concepts and speculations, the analysis accuracy is subjectively affected, even parts of image information may be neglected. Image fusion techniques provide an effective way to solve these issues [1]. As the variety of medical imaging devices increases, the obtained medical images from different modalities contain complementary as well as redundant information. Medical image fusion techniques can fuse multi-modality medical images for more reliable and accurate medical diagnosis [2,3].

This paper proposes a CNN-based medical image fusion method. First, CNN-based model generates a weight map for any-size source image. Then, Gaussian pyramid decomposition is performed on the generated weight map, and the contrast image pyramid decomposition is applied to source images for obtaining the corresponding multi-scale sub-resolution images. Next, a weighted fusion operator based on the measurement of regional characteristics is used to set different thresholds for the top layer and the remaining layers of sub-decomposed images to obtain the fused sub-decomposed images. Finally, the fused image is obtained by the reconstruction of contrast pyramid. This paper has three main contributions as follows:

(1) In training process of CNN, source images can be directly mapped to the weight map. Thus, it can also achieve the measurement of activity level and weight distribution in an optimal way to overcome the difficulties in design by learning network parameters in the training process.
(2) Human visual system is sensitive to the changes of image contrast. Thus, this paper proposes a multi-scale contrast pyramid decomposition based image fusion solution, which can selectively highlight the contrast information of fused image to achieve better human visual effects.
(3) The proposed solution uses a weighted fusion operator based on the measurement of regional characteristics. In the same decomposition layer, the fusion operators applied to different local regions may be different. Thus, the complementary and redundant information of fused image can be fully explored to achieve a better fusion effect and highlight important detailed features.

The remainder of this paper is organized as follows. Section 2 discusses the related works of medical image fusion. Section 3 demonstrates the proposed CNN-based medical image fusion solution in detail. Section 4 presents the comparative experiments and compares corresponding results. Section 5 concludes this paper.

## 2. Related Works

Researchers have proposed many medical image fusion methods in recent years [4,5]. Mainstream medical image fusion methods include decomposition-based and learning-based image fusion methods [6,7]. As a commonly used decomposition-based medical image fusion method, multi-scale transform (MST) generally has three steps in the fusion process: decomposition, fusion, and reconstruction. Pyramid-based method, wavelet, and multi-scale geometric analysis (MAG) based method are commonly used in MST [8]. In MAG-based methods, nonsubsampled contourlet transform (NSCT) [9,10] and nonsubsampled shearlet transform (NSST) [11] based methods have high efficiency in image representation. In addition to image transformation, the analysis of high- and low-frequency coefficients is also a key issue of MST-based fusion methods. Traditionally, the activity level of high-frequency coefficient is usually based on its absolute value. It is calculated in a pixel- or window-based way, and then uses a simple fusion rule, such as the selection of the maximum or weighted average, to obtain the fused coefficient. Averaging the coefficients of different source images was the most popular low-frequency fusion strategy in early research. In recent years, more advanced image transformations and more complex fusion strategies have been developed [12,13,14,15,16,17]. Liu proposed an integrated sparse representation (SR)- and MST-based medical image fusion framework [18]. Zhu proposed an NSCT based multi-modality decomposition method for medical images, which uses the phase consistency and local Laplacian energy to fuse high- and low-pass sub-bands, respectively [9]. Yin proposed a multi-modality medical image fusion method in NSST domain, which introduced pulse coupled neural network (PCNN) for image fusion [19]. To improve the fusion quality of multi-modality images, a novel multi-sensor image fusion framework based on NSST and PCNN was proposed by Li [20].

In the past decade, learning-based methods have been widely used in medical image fusion. Especially, SR- and deep learning-based fusion methods are most widely used [21,22]. In the early stage, SR-based fusion methods used a standard sparse coding model based on a single image component and local image blocks [23,24,25]. In the original spatial domain, source images were segmented into a set of overlapping image blocks for sparse coding. Most existing SR-based fusion methods attempt to improve their performances is the following ways: adding detailed constraints [5], designing more efficient dictionary learning strategy [26], using multiple sub-dictionaries in representation [27,28], etc. As an SR-based model, Kim proposed a dictionary learning method based on joint image block clustering for multi-modality image fusion. Zhu proposed a medical image fusion method based on cartoon-texture decomposition (CTD), and used an SR-based fusion strategy to fuse the decomposed coefficients [29]. Liu proposed an adaptive sparse representation (ASR) model for simultaneous image fusion and denoising [28]. All the above-mentioned methods propose complex fusion rules or different SR-based models. However, these specific rules cannot be applicable to every type of medical image fusion [27].

With the rapid development of artificial intelligence, deep learning-based image fusion methods have become a hot research topic [30,31,32,33]. As a main representative of artificial intelligence, deep learning is developed on the basis of traditional artificial neural networks. It can learn data characteristics autonomously, establish a human-like learning mechanism by simulating the neural network of human brain, and then analyze and learn the related data, such as images and texts [34,35]. CNN as a classical deep learning model can achieve the encoding of direct mapping from source images to weight map during the training process [29,36]. Thus, both activity-level measurements and weight distribution can be achieved together in an optimal way by learning network parameters. In addition, CNN’s local connection and weight sharing feature can further improve the performance of image fusion algorithms, while reducing the complexity of entire network and the number of weights. At present, CNN plays an increasingly important role in medical image fusion. Xia integrated multi-scale transform and CNN into a multi-modality medical image fusion framework, which uses the deep stacked neural network to divide source images into high- and low-frequency components to do corresponding image fusion [37]. Liu proposed a CNN-based multi-modality medical image fusion algorithm, which applies image pyramids to the medical image fusion process in a multi-scale manner [38].

The calculation of weight map, which fuses the pixel activity information from different sources, is one of the most critical issues in existing deep learning based image fusion [38]. Most existing fusion methods use a two-step solution that contains activity-level measurement and weight assignment. In traditional transform-domain fusion methods, the absolute value of decomposition coefficient is used to measure its activity first. Then, the fusion rule, such as “choose-max” or “weighted-average”, is used to select the maximum or weighted average [39]. According to the obtained measurements, the corresponding weights are finally assigned to different sources. To improve the fusion performance, many complicated decomposition methods and detailed weight assignment strategies have been proposed in recent years [28,40,41,42,43,44,45]. However, it is not easy to design an ideal activity level measurement or weight assignment strategy, which can consider all key issues [37].

## 3. The Proposed Medical Image Fusion Solution

**Figure 1:**
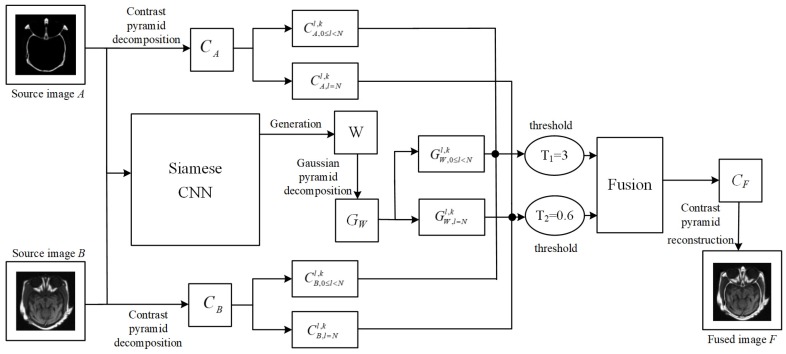
The proposed medical image fusion framework.

As shown in Figure 1, the proposed medical image fusion framework has three main steps. First, it uses Siamese network model to generate the same-size weight map *W* for any-size source image *A* and *B*, respectively. Then, Gaussian pyramid decomposition is applied to the generated weight map *W* to obtain corresponding multi-scale sub-decomposed image $G_{W}$, which is used to determine the fusion operator in coefficient fusion process. $G_{W,l=N}^{l,k}$ and $G_{W,0\leq l<N}^{l,k}$ are the top layer and the remaining layers of sub-decomposed image. It applies the contrast pyramid to the decomposition of source image *A* and *B*. The multi-scale sub-decomposed images $C_{A}$ and $C_{B}$ are obtained for the subsequent coefficient fusion process. $C_{A,l=N}^{l,k}$ and $C_{B,l=N}^{l,k}$ are the top layer of sub-decomposed image $C_{A}$ and $C_{B}$, respectively. $C_{A,0\leq l<N}^{l,k}$ and $C_{B,0\leq l<N}^{l,k}$ are used to represent the remaining layers of sub-decomposed image $C_{A}$ and $C_{B}$, respectively. Finally, different thresholds are set for the top layer and the remaining layers of sub-decomposed images, respectively. A weighted fusion operator based on the measurement of regional characteristics is used to fuse the different regions in the same decomposition layer to obtain the fused sub-decomposed image $C_{F}$. The final fused image *F* is obtained by the reconstruction of contrast pyramid.

### 3.1. Generation of CNN-Based Weight Map

#### 3.1.1. Network Construction

**Figure 2:**
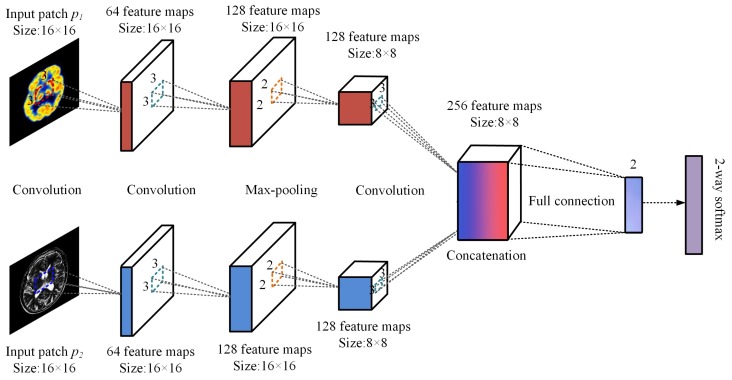
Siamese network training architecture.

To obtain the weight map of pixel activity information from multiple source images, the proposed method uses CNN to achieve the measurement of optimal pixel activity level and weight distribution. This paper uses siamese network to improve the efficiency of CNN training. Siamese network has two branches. Each branch contains three convolutional layers and one max-pooling layer. The first two layers are convolutional layers. The first layer is used for the simple feature extraction of input image. In the second layer, the number of feature maps increases. The features of output map in the upper convolutional layer are extracted. The third layer is a max-pooling layer. It removes unimportant samples from feature map to further reduce the number of parameters. As a convolution layer, the fourth layer extracts more complex features from the output map of the pooling layer. To reduce memory consumption, it uses a lightweight network structure to reduce the training complexity. Specifically, the feature map of each branch’s final output is concatenated first. Then, the concatenated ones are directly connected to a two-dimensional vector by a fully connected layer. To predict the probability distribution of different characteristics, the two-dimensional vector obtained by mapping is sent to a bi-directional softmax layer, and then classified by probability value. This paper uses the siamese network training architecture shown in Figure 2.

To achieve the classification in CNN network, this paper uses softmax classifier to obtain the classification probability by Equation (1).

(1) $$f\left(p_{i}\right)=\frac{e^{p_{i}}}{\sum_{j=1}^{n}e^{p_{j}}}$$

If one $p_{i}$ is larger than all the other *p*, then its mapping component is close to 1, and the others are close to 0, which normalizes all input vectors. The batch size is set to 128, thus the softmax loss function is obtained as Equation (2).

(2) $$L=\sum_{i=0}^{batchsize}-\log f(p_{i})$$

Taking the softmax loss function as the optimization goal, stochastic gradient descent is used to minimize the loss function. As the initial parameter settings, the momentum and weight decay are set to 0.9 and 0.0005, respectively. Thus, Equations (3) and (4) are used to update the weights.

(3) $$v_{i+1}=0.9\cdot v_{i}-0.0005\cdot \alpha \cdot w_{i}-\alpha \cdot \frac{\partial L}{\partial w_{i}}$$

(4) $$w_{i+1}=w_{i}+v_{i+1}$$

where $v_{i}$ is the dynamic variable, $w_{i}$ is weight after *i*th iteration, α is the learning rate, *L* represents the loss function, and $\frac{\partial L}{\partial w_{i}}$ is the loss derivative of weight $w_{i}$.

#### 3.1.2. Networking Training

It selects a high-quality multi-modality medical image set from http://www.med.harvard.edu/aanlib/home.html as training samples. It applies a Gaussian filter to each image to obtain corresponding five different-level fuzzy versions. Specifically, a Gaussian filter with a standard deviation of 2 and a cutoff value of 7×7 is used. Gaussian filter is used to blur the original image to obtain the first blurred image. In the following Gaussian filtering, the previous output image is used as the next input image. For instance, the output image of first Gaussian filtering is used as the input image of the second Gaussian filtering. Then, for each blurred and clear image, it randomly samples 20 pairs of 16×16 image blocks. $p_{c}$ and $p_{b}$ represent a pair of clear and blurred image blocks. When $p_{1}=p_{c}$ and $p_{2}=p_{b}$, it is defined as a positive example (marked as 1), where $p_{1}$ and $p_{2}$ are the inputs for the first and second branch, respectively. Oppositely, when $p_{1}=p_{b}$ and $p_{2}=p_{c}$, it is defined as a negative example (marked as 0). Therefore, the training set is ultimately composed of positive and negative examples. After the sample is generated, the weight of each convolutional layer is initialized by using Xavier algorithm, which adaptively determines the initialization scale based on the number of input and output neurons. The deviation of each layer is initialized to 0. The inclination rates of all layers are equal, and their initial values are set to 0.0001. When the loss reaches a steady state, the inclination rates are manually reduced to 10% of previous values. After about ten iterations, it can complete the network training.

#### 3.1.3. The Generation of Weight Map *W*

In the image testing and fusion process, to process any-size source images, it converts the fully connected layer into two equivalent convolutional layers of equal kernel size. When the conversion is completed, any-size image *A* and *B* to be fused can be processed as a whole to generate a dense prediction map *S*. Every prediction $S_{i}$ is a two-dimensional vector, and the value of each dimension is between 0 and 1. If one dimension is larger than another, this dimension can be normalized to 1, and the other one is set to 0. It simplifies the weight of corresponding image block with an output dimension value of 1. For two adjacent predictions in *S*, the steps of corresponding image blocks overlap. For overlapping areas, the weights are averaged. The output is the average weight of the overlapping image blocks. In the above way, it is possible to input any-size image *A* and *B* into the network, and generate the corresponding same-size weight map *W*.

### 3.2. Pyramid Decomposition

This paper uses both contrast pyramid and Gaussian pyramid to decompose source images. It builds the contrast pyramid first. Then, when the Gaussian pyramid is established, $G^{0}$ is the zeroth layer (bottom layer), and the *l*’th layer $G^{l}$ can be constructed in the following manner. As shown in Equation (5), it convolves $G^{l-1}$ by a window function w(m,n) with low-pass characteristics first, and then downsamples the convolutional result by the interlaced every other row and column.

(5) $$G^{l}=\sum_{m=-2}^{2}\sum_{n=-2}^{2}w\left(m,n\right)G^{l-1}\left(2i+m,2j+n\right),\;0<l\leq N,\;0\leq i<C,\;0\leq j<R_{l}$$

where w(m,n) is the window function, $C_{l}$ and $R_{l}$ are the number of columns and the number of rows in the *l*’th-layer sub-image of the Gaussian pyramid, respectively, and *N* is the total number of the pyramid layers.

(1) Separability: w(m,n)=w(m)w(n),$m\in \left[-2,2\right],n\in \left[-2,2\right];$
(2) Normalization: $\sum_{m=-2}^{2}w(n)=1;$
(3) Symmetry: w(n)=w(−n); and
(4) Equal contribution of odd and even terms: w(−2)+w(2)+w(0)=w(−1)+w(1).

According to the above constraints, it can construct w(0)=3/8, w(1)=w(−1)=1/4, and w(2)=w(−2)=1/16. Then, according to Constraint 1, it can get the window function w(m,n) by calculation, as shown in Equation (6).

(6) $$w=\frac{1}{256}\left[\begin{array}{ccccc} 1 & 4 & 6 & 4 & 1\\ 4 & 16 & 24 & 16 & 4\\ 6 & 24 & 36 & 24 & 6\\ 4 & 16 & 24 & 16 & 4\\ 1 & 4 & 6 & 4 & 1 \end{array}\right].$$

At this point, the image Gaussian pyramid is constructed by $G^{0},G^{1},\cdots ,G^{N}$.

After the construction of a Gaussian pyramid image by halving the size of each layer one by one, the interpolation method is used to interpolate and expand the Gaussian pyramid. Thus, the expanded *l*th-layer image $G^{l}$ and the l−1th-layer image $G^{l-1}$ have the same size and the operation is shown as follows:

(7) $$G_{*}^{l}\left(i,j\right)=4\sum_{m=-2}^{2}\sum_{n=-2}^{2}w\left(m,n\right)G^{l}\left[\frac{m+i}{2},\frac{n+j}{2}\right],0<l\leq N,0<i<C_{l},0<j<R_{l}$$

(8) $$\begin{array}{c} G^{l}\left[\frac{m+i}{2},\frac{n+j}{2}\right]=\left\{\begin{array}{c} G^{l}\left(\frac{m+i}{2},\frac{n+j}{2}\right),\begin{array}{cc} & \mathrm{when}\;\frac{m+i}{2},\frac{n+j}{2}\;\mathrm{are}\;\mathrm{integer} \end{array}\\ \begin{array}{cc} 0, & \;\mathrm{otherwise} \end{array} \end{array}\right. \end{array}$$

where $G_{*}^{l}$ is an expansion version of image Gaussian pyramid $G^{l}$. According to the above formulas, an expansion sequence is obtained by interpolating and expanding each layer of Gaussian pyramid, respectively.

According to the above formulas, an expansion sequence is obtained by interpolating and expanding each layer of Gaussian pyramid respectively. The decomposition of image contrast is shown as Equation (9).

(9) $$\begin{array}{c} C^{l}=\frac{G^{l}}{G_{*}^{l}}-I,\begin{array}{ccc} & & N>l\geq 0 \end{array}\\ C^{N}=G^{N},\begin{array}{ccc} & & l=N \end{array} \end{array}$$

where $C^{l}$ is the contrast pyramid, $G^{l}$ is the Gaussian pyramid, *I* is the image decomposed by contrast pyramid, and *l* is the decomposition level, which composes the contrast pyramid $C^{0},C^{1},\cdots ,C^{N}$ of source image.

Source image *A* and *B* are decomposed into corresponding sub-images by contrast pyramid, respectively. For the weight map generated in CNN network, it is decomposed into sub-images by Gaussian pyramid. Different thresholds are set for the top layer and the remaining layers of the obtained sub-images respectively in the fusion processing.

### 3.3. Fusion Rules

In the fusion process, to obtain better visual characteristics, richer details, and outstanding fusion effects, this paper adopts new fusion rules and the weighted average fusion operators based on regional characteristics. The fusion rules and operators are shown as follows:

(1) After the contrast pyramid decomposition, it calculates the energy $E_{A}^{l}$ and $E_{B}^{l}$ of corresponding local regions in each decomposition level *l* of source image *A* and *B*, respectively.
  (10) $$\begin{array}{c} E_{A}^{l}\left(x,y\right)=\sum_{m}\sum_{n}C_{A}^{l}{\left(x+m,y+n\right)}^{2}\\ E_{B}^{l}\left(x,y\right)=\sum_{m}\sum_{n}C_{B}^{l}{\left(x+m,y+n\right)}^{2} \end{array}$$
  where $E^{l}\left(x,y\right)$ represents the local area energy centered at (x,y) on the *l*th layer of contrast pyramid, $C_{}^{l}$ is the *l*th-layer image of contrast pyramid, and *m* and *n* represent the size of local area.
(2) Calculate the similarity of corresponding local regions in two source images.
  (11) $$M^{l}\left(x,y\right)=\frac{2\sum_{m}\sum_{m}C_{A}^{l}\left(x+m,y+n\right)C_{B}^{l}\left(x+m,y+n\right)}{E_{A}^{l}\left(x,y\right)E_{B}^{l}\left(x,y\right)}$$
  where $E_{A}^{l}$ and $E_{B}^{l}$ are calculated by Equation (10). The range of similarity is [–1,1], and a value close to 1 indicates high similarity.
(3) Determine the fusion operators. Define a similarity threshold *T* (when 0≤l<N, $T_{1}=3$; when l=N, $T_{2}=0.6$). When $M^{l}\left(x,y\right)<T$, it obtains:
  (12) $$\left.\begin{array}{c} \mathrm{when}\;E_{A}^{l}\left(x,y\right)\geq E_{B}^{l}\left(x,y\right),C_{F}^{l}\left(x,y\right)=C_{A}^{l}\left(x,y\right);\\ \mathrm{when}\;E_{A}^{l}\left(x,y\right)<E_{B}^{l}\left(x,y\right),C_{F}^{l}\left(x,y\right)=C_{B}^{l}\left(x,y\right); \end{array}\right\}$$
  when $M^{l}\left(x,y\right)\geq T$, weight map *W* based weighted mean model is:
  (13) $$\left.\begin{array}{c} \mathrm{when}\;E_{A}^{l}\left(x,y\right)\geq E_{B}^{l}\left(x,y\right),\\ C_{F}^{l}\left(x,y\right)=W_{\max}^{l}\left(x,y\right)C_{A}^{l}\left(x,y\right)+W_{\min}^{l}\left(x,y\right)C_{B}^{l}\left(x,y\right);\\ \mathrm{when}\;E_{A}^{l}\left(x,y\right)<E_{B}^{l}\left(x,y\right),\\ C_{F}^{l}\left(x,y\right)=W_{\min}^{l}\left(x,y\right)C_{A}^{l}\left(x,y\right)+W_{\max}^{l}\left(x,y\right)C_{B}^{l}\left(x,y\right); \end{array}\right\}$$
  where $C_{F}^{l}$ is the *l*th layer of sub-image after fusion.
  (14) $$\begin{array}{c} W_{\min}^{l}\left(x,y\right)=G_{W}^{l}\left(x,y\right)\\ W_{\max}^{l}\left(x,y\right)=1-W_{\min}^{l}\left(x,y\right) \end{array}$$
  Finally, the integration strategy can be summarized as a whole by Equation (15).
  (15) $$C_{F}^{l}\left(x,y\right)=\left\{\begin{array}{c} C_{A}^{l}\left(x,y\right),\\ if\;M^{l}\left(x,y\right)<T\&E_{A}^{l}\left(x,y\right)\geq E_{B}^{l}\left(x,y\right);\\ C_{B}^{l}\left(x,y\right),\\ if\;M^{l}\left(x,y\right)<T\&E_{A}^{l}\left(x,y\right)<E_{B}^{l}\left(x,y\right);\\ W_{\max}^{l}\left(x,y\right)C_{A}^{l}\left(x,y\right)+W_{\min}^{l}\left(x,y\right)C_{B}^{l}\left(x,y\right),\\ if\;M^{l}\left(x,y\right)\geq T\&E_{A}^{l}\left(x,y\right)\geq E_{B}^{l}\left(x,y\right);\\ W_{\max}^{l}\left(x,y\right)C_{A}^{l}\left(x,y\right)+W_{\min}^{l}\left(x,y\right)C_{B}^{l}\left(x,y\right),\\ if\;M^{l}\left(x,y\right)\geq T\&E_{A}^{l}\left(x,y\right)\geq E_{B}^{l}\left(x,y\right); \end{array}\right.$$

According to the above algorithm, when the similarity between the corresponding local regions of source image *A* and *B* is less than threshold *T*, it means that the “energy” difference of two local regions is large. At this time, the central pixel of the region with a larger “energy” is selected as the central pixel of corresponding region in the fused image. Conversely, when the similarity is greater than or equal to threshold *T*, it means that the “energy” of the region is similar in two source images. At this time, the weighted fusion operator is used to determine the contrast or gray value of the central pixel of the region in the fused image.

Since the central pixel with large local energy represents a distinct feature of source image, the local image features generally do not only depend on a certain pixel. Therefore, the weighted fusion operator based on region characteristics is used, which is more reasonable than other determination methods of fused pixel based on the simple selection or the weight of an independent pixel.

Finally, the decomposed sub-image $C_{F}^{l}$ obtained after fusion is inversely transformed by contrast pyramid, which is also called image reconstruction. According to Equation (16), the accurate image reconstruction by contrast pyramid can be obtained.

(16) $$\begin{array}{cc} G^{l}=\left(C^{l}+F\right)⊙G_{*}^{l}, & N>l\geq 0\\ G^{N}=C^{N}, & l=N \end{array},$$

where ⊙ denotes Hadamard product ( also known as the element-wise multiplication ).

The fused image *F* can be obtained by calculating the above-mentioned image reconstruction formula. Algorithm 1 shows the main steps of the proposed medical image fusion solution.
**Algorithm 1** Proposed NSST-based multi-sensor image fusion framework.**Input:**        source image *A* and *B*;        Parameters: pyramid decomposition level *l*, the number of pyramid levels *N*, similarity threshold *T* **Output:**        the fused image *F*
1:It inputs two any-size source images *A* and *B* to the trained siamese network.2:It generates a dense prediction map *S*, where each prediction has two dimensions.3:**for** any prediction $S_{i}$ **do**4:    It does normalization processing to obtain a corresponding image block weight with a dimension    value of 1.5:**end for**6:**for** an overlapping region of two adjacent predictions $S_{j}$ and $S_{j+1}$ **do**7:    It does the averaging process to obtain the mean value of the overlapping image block weights.8:    It outputs the same size weight map *W* as source image.9:**end for**10:**for** each source image *A*, *B*, and weight map *W* **do**11:    It does pyramid decomposition respectively to obtain a contrast sub-images $C_{A}^{}$, $C_{B}^{}$ and a    Gaussian sub-image $G_{W}^{}$.12:    **for** each decomposition level *l* obtained by the contrast pyramid decomposition of source image    **do**13:        It calculates the energy $E_{A,B}^{l}\left(x,y\right)$ of its corresponding local area.14:        It determines the similarity of fusion mode $M^{l}\left(x,y\right)$.15:        It defines a similarity threshold *T* (when 0≤l<N, $T_{1}$=3; when l=N, $T_{2}$=0.6) to determine        the strategy of coefficient fusion.16:    **end for**17:**end for**18:The fused image *F* is obtained by the inverse pyramid transform of sub-image $C_{F}^{l}$ after fusion.


## 4. Comparative Experiments and Analysis

### 4.1. Experiment Results and Analysis

The following comparative experiments were used to prove that the proposed CNN-based algorithm has good performance in medical image fusion. Eight different image fusion methods were used to fuse MR-CT, MR-T1-MR-T2, MR-PET, and MR-SPECT images, respectively. These eight methods are MST-SR [18], NSCT-PC [9], NSST-PCNN [19], ASR [28], CT [29], KIM [26], CNN-LIU [38], and the proposed solution.

**Figure 3:**
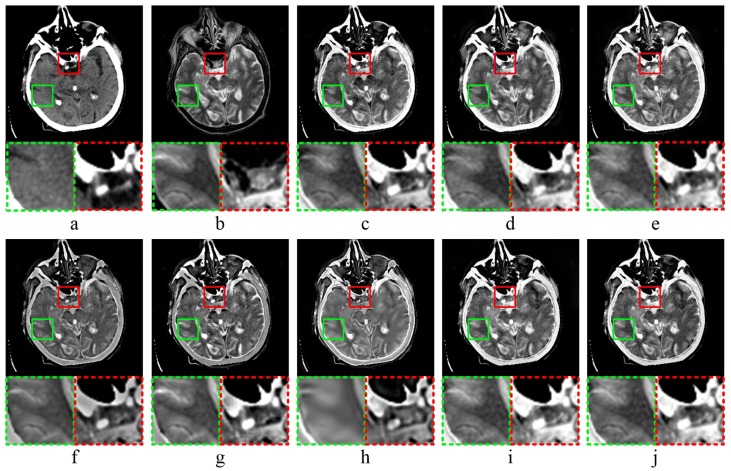
MR-CT image fusion experiments: (**a**,**b**) source images; and (**c**–**j**) the fused image obtained by MST-SR, NSCT-PC, NSST-PCNN, ASR, CT, KIM, CNN-LIU, and the proposed method, respectively. Two partially enlarged images marked in green and red dashed frames correspond to the regions surrounded by green and red frames in the fused image.

Figure 3 shows the results of MR-CT image fusion experiments. In Figure 3c, the fused image obtained by MST-SR method has a general visualization performance, and the image contrast is high by analyzing the partially enlarged image. As shown in Figure 3d,e, the fused images by NSCT-PC and NSST-PCNN have a high brightness. According to the partial enlargements marked in green and red dashed frame, both methods have the poor performance in the preservation of image details. In Figure 3f,g, the fused images obtained by ASR and CT have low brightness. According to the analysis of details, the detailed information of image edge is not obvious, which is not good for human eye observation. As shown in Figure 3h, the fused image obtained by KIM has low sharpness and poor visual effect. Comparing Figure 3i,j, as well as the partially magnified images, it is difficult to visually distinguish the quality of the fused image obtained by CNN-LIU and the proposed method.

**Figure 4:**
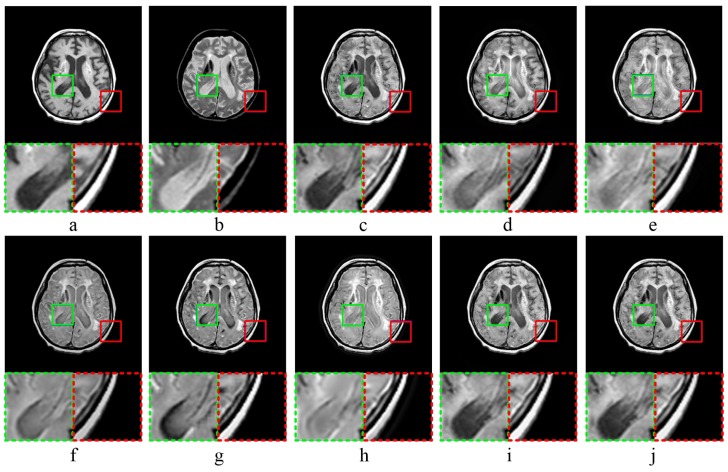
MR-T1 CMR-T2 image fusion experiments: (**a**,**b**) source images; and (**c**–**j**) the fused image obtained by MST-SR, NSCT-PC, NSST-PCNN, ASR, CT, KIM, CNN-LIU, and the proposed method, respectively. Two partially enlarged images marked in green and red dashed frames correspond to the regions surrounded by green and red frames in the fused image.

The results of MR-T1-MR-T2 image fusion experiments are shown in Figure 4. Comparing the fused result (Figure 4c) with source image (Figure 4a,b), the fused image obtained by MST-SR method has a low similarity to source image (Figure 4a), and does not well retain the detailed structure information of source image (Figure 4a). As shown in Figure 4d, the fused image obtained by NSCT-PC method is too smooth in some areas, and the detailed image texture is not sufficiently obvious. In Figure 4e, the fused image obtained by NSST-PCNN method has high brightness, and does not well preserve the detailed features of source images. ASR method obtains the fused image with low contrast and a lot of noises, as shown in Figure 4f. The fused image shown in Figure 4g was obtained by CT method, and has high edge brightness, which weakens the detailed texture information of image edges. According to Figure 4h, the fused image obtained by KIM method has low sharpness, and is blurred. As shown in Figure 4i,j, CNN-LIU and the proposed method reach the almost same visual performance of human eyes.

**Figure 5:**
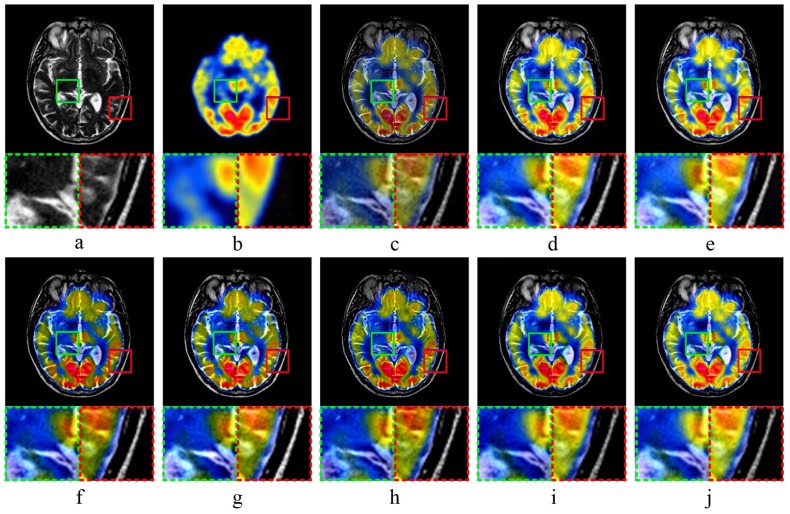
MR-PET image fusion Experiment 1: (**a**,**b**) source images; and (**c**–**j**) the fused image obtained by MST-SR, NSCT-PC, NSST-PCNN, ASR, CT, KIM, CNN-LIU, and the proposed method, respectively. Two partially enlarged images marked in green and red dashed frames correspond to the regions surrounded by green and red frames in the fused image.

**Figure 6:**
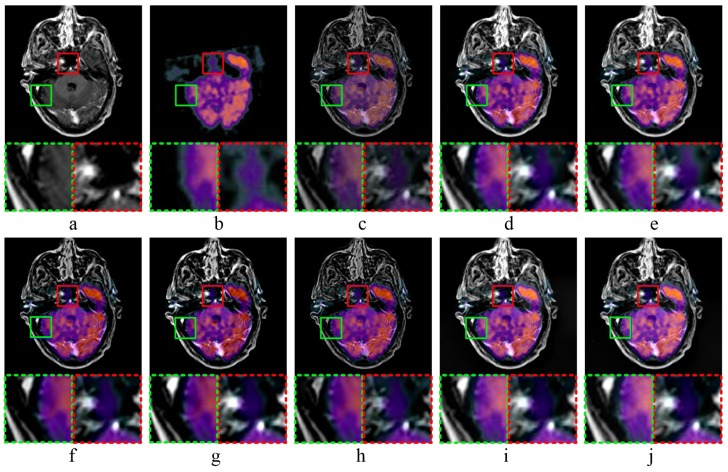
MR-PET image fusion Experiment 2: (**a**,**b**) source images; and (**c**–**j**) the fused image obtained by MST-SR, NSCT-PC, NSST-PCNN, ASR, CT, KIM, CNN-LIU, and the proposed method, respectively. Two partially enlarged images marked in green and red dashed frames correspond to the regions surrounded by green and red frames in the fused image.

Figure 5 and Figure 6 show the results of MR-PET image fusion experiments. In both Figure 5c and Figure 6c, the fused images obtained by MST-SR method have high darkness, which is not conducive to human visual observation. According to Figure 5d,e, as well as the partially magnified areas, the fused images obtained by NSCT-PC and NSST-PCNN method have high brightness, and the detailed image information is not clear. Figure 5f,g and Figure 6f,g show that the fused images obtained by ASR and CT methods have low brightness. It means that these two methods have poor performance in the preservation of image details. Comparing Figure 5i,j, the fused image of KIM method has low sharpness, which means the image is blurred. As shown in Figure 5i, the fused image obtained by CNN-LIU method has a low contrast, and the detailed edge information is not obvious. Both Figure 5j and Figure 6j show that the proposed fusion method can preserve the detailed information of source images well, which is conducive to the observation of medical images and the diagnosis of diseases.

**Figure 7:**
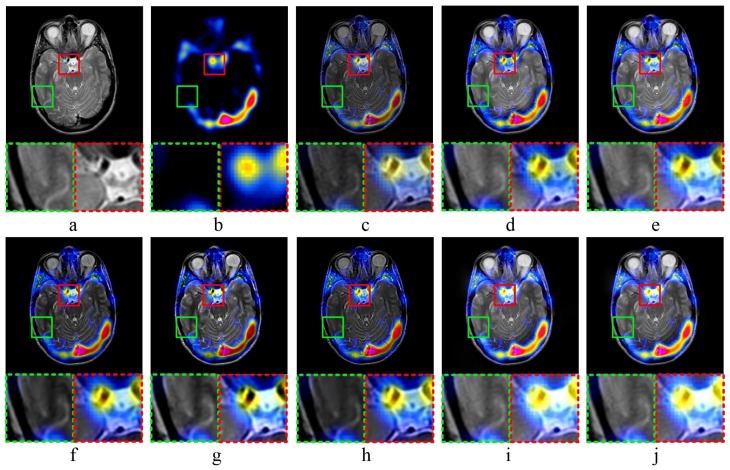
MR-SPECT image fusion Experiment 1: (**a**,**b**) source images; and (**c**–**j**) the fused image obtained by MST-SR, NSCT-PC, NSST-PCNN, ASR, CT, KIM, CNN-LIU, and the proposed method, respectively. Two partially enlarged images marked in green and red dashed frames correspond to the regions surrounded by green and red frames in the fused image.

**Figure 8:**
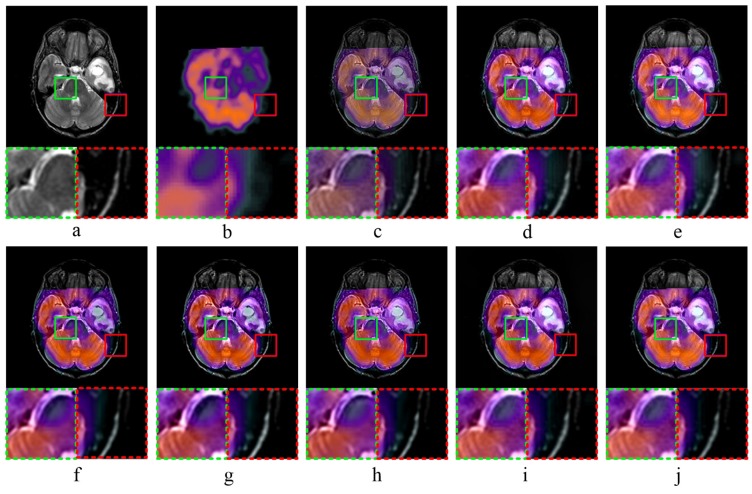
MR-SPECT image fusion Experiments 2: (**a**,**b**) source images; and (**c**–**j**) the fused image obtained by MST-SR, NSCT-PC, NSST-PCNN, ASR, CT, KIM, CNN-LIU, and the proposed method, respectively. Two partially enlarged images marked in green and red dashed frames correspond to the regions surrounded by green and red frames in the fused image.

Figure 7 and Figure 8 show the results of MR-SPECT image fusion experiments. In Figure 7c, the fused image of MST-SR method has a low contrast and unclear edge details. As shown in Figure 8d,e, some edge regions are too smooth in the fused images obtained by NSCT-PC and NSST-PCNN methods, and the edge details are not clear. In Figure 7g and Figure 8g, the images obtained by CT method have the high contrast, and CT method performs poorly on the detail retention of source images. The fused images shown in Figure 7f,h and Figure 8f,h, which were obtained by ASR and KIM method, respectively, have the low brightness and poor visualization performance. As shown in Figure 7i,j and Figure 8i,j, the fused images obtained by both CCN-LIU and the proposed method have the high brightness and good visualization performance. Comparing all the fused results in Figure 7 and Figure 8, the fused images obtained by the proposed fusion method have the high similarity with source images, which can preserve the detailed structures of source images well and achieve good fusion performance.

### 4.2. Evaluation of Objective Metrics

**Figure 9:**
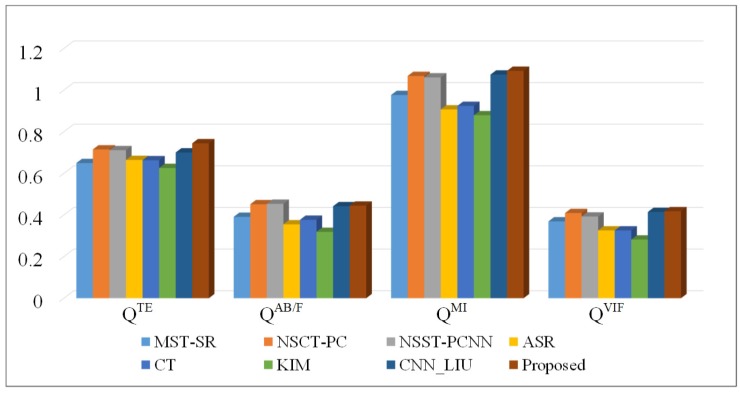
Objective evaluation results of eight fusion methods.

For image fusion, a single evaluation metric lacks objectivity. Therefore, it is necessary to do a comprehensive analysis by using multiple evaluation metrics. In this study, four objective evaluation metrics, namely $Q^{TE}$ [46,47], $Q^{AB/F}$ [29,48], $Q^{MI}$ [47], and $Q^{VIF}$ [29,49], were used to evaluate the performances of different fusion methods. $Q^{TE}$ is the Tsallis entropy of the fused image. The entropy value represents the amount of average information contained in the fused image. $Q^{AB/F}$ as a gradient-based quality indicator is mainly used to measure the edge information of fused images. $Q^{MI}$ is the mutual information indicator, which is used to measure the amount of information contained in the fused image. $Q^{VIF}$ is the information ratio between the fused image and source images to evaluate the human visualization performance of the fused image. The objective evaluation results of medical image fusion are shown in Figure 9. Among all the fusion results, the proposed method achieves good performance in all four objective evaluations. It confirms that the proposed method can preserve the detailed structure information of source images well and realize good human visual effects.

Table 1 shows the values of four objective metrics for eight fusion methods. The proposed method achieves the highest $Q^{TE}$ value. Comparing with the seven other fusion methods, the fused image obtained by the proposed method has the highest Tsallis entropy, and contains more information than the others. According to the analysis of $Q^{AB/F}$, the fused images obtained by NSCT-PC, NSST-PCNN, CNN-LIU, and the proposed method have high $Q^{AB/F}$, which means these fused images perform well in the preservation of edge details. The fused image obtained by KIM has low $Q^{AB/F}$, which indicates that KIM does not have good performance in the preservation of edge information. For $Q^{MI}$, the proposed method is a little bit higher than the others. It means more information of source images is retained in the fused image, and the preservation ability of source image details is strong. The proposed method has the highest $Q^{VIF}$. Comparing with CNN-LIU, the proposed method has a higher information ratio between the fused image and source images, and achieves a better human visual effect as well.

### 4.3. Threshold Discussion

In this study, a similarity threshold *T* was defined to fuse the multi-scale sub-decomposed images. For the top layer of sub-decomposed images, the threshold was set to 0.6. For the remaining layers of sub-decomposed images, the threshold was set to 3. Table 2 shows the values of five objective metrics for the fusion framework with different thresholds. According to $Q^{MI}$, the proposed method is a little bit lower than others. However, for $Q^{TE}$, $Q^{AB/F}$, and $Q^{VIF}$, the proposed method is higher than the others. It means more average information and edge information is contained in the fused image, and it has a higher information ratio between the fused image and source images. In addition, these three methods have close values in terms of time consumption. Overall, the proposed method performs better on five objective metrics.

## 5. Conclusions

This paper proposes a CNN-based medical image fusion solution. The proposed method implements the measurement of activity level and weight distribution by CNN training to generate a weight map including the integrated pixel activity information. To obtain better visual effects, the multi-scale decomposition method based on contrast pyramid is used to fuse corresponding image components in different spatial frequency bands. Meanwhile, the complementary and redundant information of fused images is explored by the local similarity strategy in adaptive fusion mode. Comparative experiment results show that the fused images by proposed method have high visual quality and objective indicators. In the future, we will continue to explore the great potential of deep learning techniques and apply them to other types of multi-modality image fusion, such as infrared-visible and multi-focus image fusion.

## Figures and Tables

**Table 1:** Objective evaluations of medical image fusion comparative experiments.

	$Q^{\mathrm{TE}}$	$Q^{\mathrm{AB}/F}$	$Q^{\mathrm{MI}}$	$Q^{\mathrm{VIF}}$	Average Processing Time
MST-SR	0.6495	0.3911	0.9764	0.3693	15.0541
NSCT-PC	0.7150	0.4515	1.0681	0.4092	3.7743
NSST-PCNN	0.7113	**0.4537**	1.0610	0.3924	6.1595
ASR	0.6643	0.3550	0.9072	0.3258	35.2493
CT	0.6631	0.3769	0.9240	0.3258	14.5846
KIM	0.6257	0.3188	0.8792	0.2820	59.1929
CNN-LIU	0.7003	0.4421	1.0745	0.4145	14.5846
Proposed	**0.7445**	0.4449	**1.0925**	**0.4181**	12.8667

**Table 2:** Objective evaluations of the fusion framework with different thresholds.

	$Q^{\mathrm{TE}}$	$Q^{\mathrm{AB}/F}$	$Q^{\mathrm{MI}}$	$Q^{\mathrm{VIF}}$	Average Processing Time
Threshold = 0.6	0.6973	0.4248	1.1165	0.4151	12.7256
Threshold = 3	0.7289	0.4255	**1.2540**	0.4068	12.6652
Proposed	**0.7445**	**0.4449**	1.0925	**0.4181**	12.8667
